# Identification of *PFKFB2* as a key gene for the transition from acute to old myocardial infarction in peripheral blood

**DOI:** 10.3389/fcvm.2022.993579

**Published:** 2022-12-06

**Authors:** Xiangyu Yang, Jie Li, Xinyao Hu, Yinzhuang Zhang, Yuanyuan Kuang, Yubo Liu, Chenxi Liu, Haodong Gao, Li Ma, Jia Tang, Qilin Ma

**Affiliations:** ^1^Department of Cardiology, Xiangya Hospital, Central South University, Changsha, China; ^2^National Clinical Research Center for Geriatric Disorders, Xiangya Hospital, Central South University, Changsha, China; ^3^Department of Reproductive Medicine, Tongji Hospital, Tongji Medical College, Huazhong University of Science and Technology, Wuhan, China

**Keywords:** myocardial infarction, immune cells, *PFKFB2*, energy metabolism, glycolysis

## Abstract

**Objective:**

This study aims to analyze the gene expression profile of peripheral blood in different stages of myocardial infarction (MI) by transcriptome sequencing, and to study the gene expression characteristics of peripheral blood after MI.

**Methods:**

Differentially expressed genes (DEGs) and weighted gene co-expression network analysis (WGCNA) were used to identify genes and modules associated with old myocardial infarction (OMI). Gene Ontology (GO) functional annotation and Kyoto Encyclopedia of Genes and Genomes (KEGG) pathway annotation were applied to analyze the potential functions of genes. Hub genes were identified by Random Forest Classifier. CIBERSORT was used to provide an estimate of the abundance of 22 immune cells in peripheral blood. Quantitative polymerase chain reaction (qPCR) was used to detect gene expression levels in clinical samples. The cellular components (CC) of peripheral blood were counted by an automatic hematology analyzer.

**Results:**

Through differential gene analysis and co-expression network analysis, 11 candidate genes were obtained. A random forest classifier identified 10 hub genes. Immune cell distribution of peripheral blood was found that T cell CD4 memory resting, NK cells resting, Dendritic cells activated, Mast cells resting, Monocytes and Neutrophils were correlated with OMI. Spearman correlation analysis found that *PFKFB2* is related to the above immune cells. Low expression of *PFKFB2* in peripheral blood of OMI was detected in clinical samples, and the relationship between *PFKFB2* and peripheral blood immune cell counts was analyzed, which showed monocytes were associated with *PFKFB2* in our study.

**Conclusion:**

*PFKFB2* was low expressed in OMI, and related to the distribution of immune cells. *PFKFB2* may play a key role in reflecting the transition from AMI to OMI, and predicting the distribution of immune cells, which provided a new perspective for improving myocardial fibrosis and adverse remodeling.

## Introduction

Myocardial infarction (MI) is one of the major adverse cardiovascular events in the world, with high morbidity and mortality (1). The number of MI cases in the world is about 7.29 million every year (2). At present, a relatively complete diagnosis and treatment strategy for MI has been developed. The mortality of patients with acute MI (AMI) have been significantly decreased (3), and progressing to old myocardial infarction (OMI) becomes the most common outcome. As a long-term pathophysiological stage, OMI inevitably occurs in ventricular remodeling and myocardial fibrosis, leading to the occurrence of adverse events such as heart failure, which influences the quality of life of patients with the disease (4). However, we still lack awareness of this process.

Peripheral blood-related biomarkers have been widely used for early diagnosis and evaluation after AMI. Cardiac troponin I (*cTnI*) and T (*cTnT*) are the preferred biomarkers for clinical evaluation of the myocardial injury, and their peripheral blood levels are highly correlated with infarct size (5). Galectin-3 and Suppression of Tumorigenicity (*ST2*) are up-regulated in the peripheral blood of patients with AMI, and they are used as prognostic markers for MI (6, 7). Therefore, we aimed to explore the differential gene expression in peripheral blood in different stages of MI (AMI and OMI), analyze the characteristics of genes related to the development from AMI to OMI, and find potential targets for early clinical intervention of AMI.

In this study, we combined weighted gene co-expression network analysis (WGCNA) and Random Forest Model to obtain key genes. Then we further used Cibersort to find the differential distribution of immune cells in the peripheral blood of AMI and OMI and found that PFKFB2 is related to the differential distribution of immune cells. Finally, PFKFB2 was considered to be a key gene for reflecting the transition and predicting the level of immune cells from AMI to OMI, which may be a potential target providing a rationale for improving MI outcomes.

## Materials and methods

### Data collection

The data used in this study was from GSE123342. It preserved the results of peripheral blood mRNA sequencing of AMI patients in a prospective trial, including AMI (D0, *n* = 65), 30 days post-MI (D30, *n* = 64), 1–year post-MI (Y1, *n* = 37), patients with stable CAD (SCAD, *n* = 22) and technical replicates (*n* = 4). Inclusion and exclusion criteria were described by Vanhaverbeke et al. (8). In short, patients diagnosed with type I MI with or without ST-elevation were included, and patients with pre-existing inflammatory disease or cardiomyopathy, Killip class III-IV or renal failure were excluded. AMI (D0, *n* = 67, including two technical sample duplications) and 1–year post-MI (Y1, *n* = 37), namely AMI and OMI, are performed in downstream analysis (Supplementary Table 1).

### Data preprocessing and study design

**FIGURE 1:**
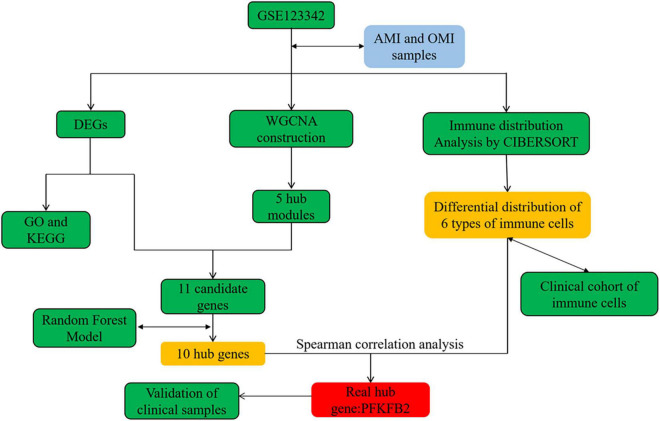
The flow chart of the study design and analysis.

Raw data were preprocessed on the Affymetrix Expression Console 1.4.1.46 and normalized using Robust Multi-Array Average and Signal Space Transformation as described by Vanhaverbeke et al. (8). The processed data was used directly in our analysis. The flow chart of this study was in Figure 1.

### Differentially expressed genes and enrichment analysis

The R package “limma” (version 3.50.0) was used to identify DEGs between AMI and OMI (9). An adjusted *P-*value < 0.05 and | log2 fold change| > 1 were used as cut-off values. The R package “clusterProfiler” (version 4.2.1) was used for GO enrichment and KEGG analysis (10). Adjust *p-*value < 0.05 and false discovery rate (FDR) < 0.05 were considered statistically significant.

### Gene set enrichment analysis

We perform Gene set enrichment analysis (GSEA) using the MSigDB KEGG database. We then filtered the results of GSEA (*p-*value < 0.05, FDR < 0.2).

### Construction of co-expression network

We selected a total of top 5,000 genes with median absolute deviation, and then constructed the co-expression network by “WGCNA” package (version 1.70-3) (11). When the correlation coefficient threshold was 0.85, we chose the soft thresholding power to be 5. We set 0.25 as the threshold for merging possible similar modules. The modules related to OMI were selected.

### Screening candidate genes

We obtained 11 candidate genes from the intersection of DEGs and genes within the clinical trait association modules. The “randomForest” package (version 4.7–1.1) was applied to build a random forest model based on these 11 candidate genes, and selected genes with Gini coefficients greater than 2 as our hub genes. Then we calculated the spearman correlation coefficient matrix of hub genes and 6 types of immune cells to determine immune-related genes.

### Evaluation of the distribution of immune cells in peripheral blood

In this study, we used the “CIBERSORT,” a classic tool for the analysis of immune cell distribution, to estimate the fraction of 22 types of immune cells (11). It can evaluate the abundance of immune cells in bulk RNA-sequencing matrix by deconvolution.

### Patients and clinical information

According to the inclusion and exclusion criteria described above, we included patients admitted to the Cardiology Department of Xiangya Hospital in May 2022: AMI group (*n* = 15) and OMI group (*n* = 18). All OMI patient were diagnosed according to Thygesen et al. (12). We collected the peripheral blood of these patients within 24 h after admission, and obtained the results of the first blood routine examination from the medical records. The study was approved by the Ethics Committee of Xiangya Hospital, informed consent was obtained from the patients, and it complied with the principles outlined in the Declaration of Helsinki. In addition, under the above inclusion and exclusion criteria, we also collected patients with unstable angina (UA) and no previous history of MI (UA, *n* = 9) for subsequent discussion.

### Real-time quantitative polymerase chain reaction

We extracted and purified total RNA from blood samples using the NucleoSpin RNA Blood Mini Kit (MACHEREY-NAGEL, Germany) according to the manufacturer’s protocol. Purity and integrity qualified total RNA was transcribed into cDNA using the HiScript II First Strand Synthesis Kit (Vazyme Bio, China). We performed quantitative polymerase chain reaction (qPCR) using SYBR qPCR Master Mix (Vazyme Bio, China). The primer sequence (5′->3′) for *GAPDH* are forward primer: AGGTCCACCACTGACACGTT; reverse primer: GCCTCAAGATCATCAGCAAT. The primer sequence (5′->3′) for *PFKFB2* are forward primer: CCTCAGAAC AGAACAACAACAG; reverse primer: TGAGGTAGCGTG TTAGTTTCTT. Expression levels of *PFKFB2* were expressed as fold changes relative to expression levels of *GAPDH* and were calculated by the 2-ΔΔCt method.

### Statistical analysis

**FIGURE 2:**
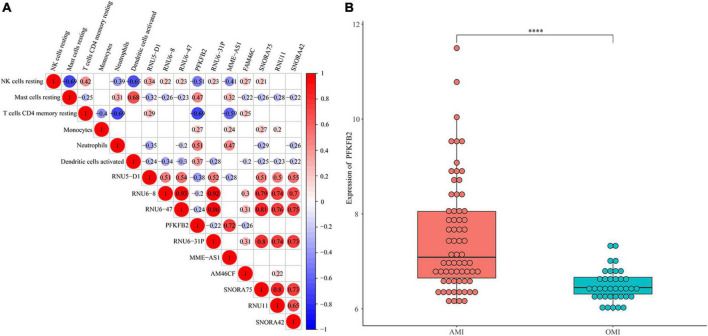
Identification of immune-related gene. **(A)** Spearman correlation analysis of 10 genes and 5 immune cells. The bigger the circle size, the more correlative (*P* < 0.05). Blank cells indicate no correlation. **(B)** Expression levels of *PFKFB2* between AMI and OMI samples. Displayed with normalized values. ^****^P < 0.0001.

**FIGURE 3:**
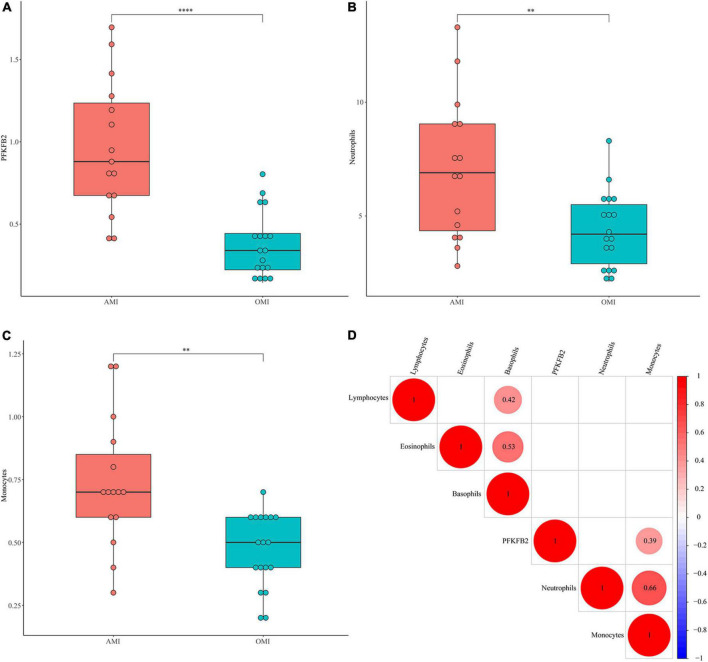
Validation of *PFKFB2* and immune cells in clinical samples. **(A)** The expression levels of *PFKFB2* in peripheral blood of AMI and OMI patients were detected by qPCR. **(B,C)** The distribution of neutrophil counts, basophil counts, monocyte counts, lymphocyte counts, and eosinophil counts in AMI and OMI is shown, respectively, (1 × 10^9^). **(D)** Spearman correlation analysis of *PFKFB2* and peripheral blood immune cells. The bigger the circle size, the more correlative (*P* < 0.05). Blank cells indicate no correlation. ^**^P < 0.01, ^****^P < 0.0001.

**FIGURE 4:**
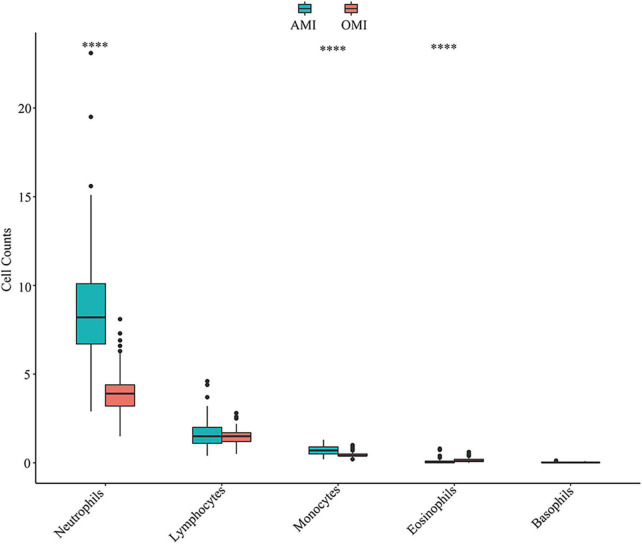
Distribution of immune cells in peripheral blood of AMI and OMI in clinical cohort. ^****^P < 0.0001.

The statistical significance of differences between the two groups in Figures 2–4 was analyzed using Wilcoxon test. *P-*value less than 0.05 was considered significant. All analyses were conducted using software R 4.1.2.

## Results

### Identification of differentially expressed genes and enrichment analysis in peripheral blood transcriptome of acute MI and old myocardial infarction

**FIGURE 5:**
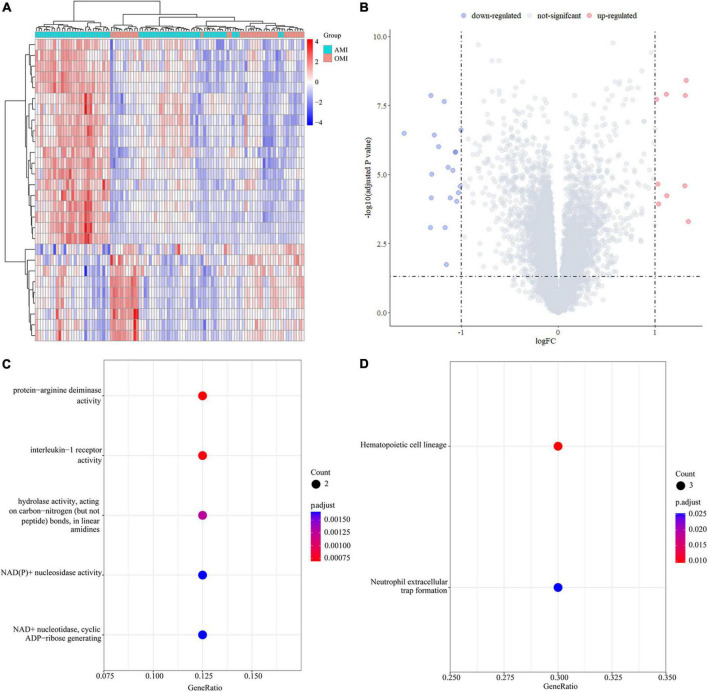
Identification of DEGs in the GSE123342 database. **(A)** Heatmap of DEGs in 104 samples between AMI (D0, *n* = 67) and OMI (Y1, *n* = 37). **(B)** Volcano plot of differential expression analysis results. The abscissa is logFC and the ordinate is –log10 p. adj. The (upper right) part has a p. adj less than 0.05 and a fold change greater than 2, indicating significant DEGs with higher expression levels. The (upper left) part has a p. adj less than 0.05 and a fold change less than −2, indicating significant DEGs with reduced expression. **(C)** GO enrichment of DEGs. Biological processes (BP) are shown on the (top), CC are shown in the middle, and MF is shown at the (bottom). **(D)** KEGG enrichment of DEGs.

We calculated 28 DEGs (9 upregulated and 19 downregulated) in AMI and OMI by the “limma” package (Figures 5A,B). GO analysis of the 26 DEGs showed that genes were mainly involved in molecular functions (MF) associated with energy metabolism (Figure 5C). At the same time, the results of KEGG manifested changes in immune regulation (Figure 5D). These results suggested that both energy metabolism and immune regulation were altered during the transition from AMI to OMI.

### Identification of key modules in the co-expression network

**FIGURE 6:**
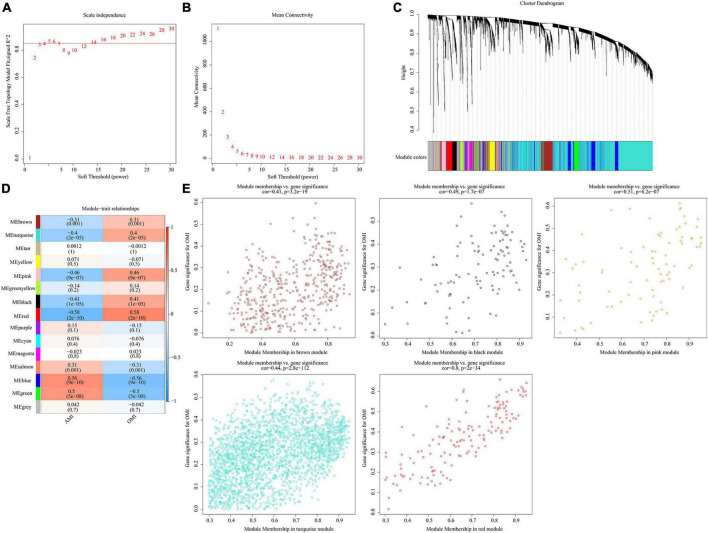
Identification of key modules in the co-expression network. **(A)** Analysis of the scale-free fit index. **(B)** Analysis of the mean connectivity for various soft-thresholding powers. **(C)** Clustering dendrograms, with dissimilarity based on topological overlap, together with assigned module colors. **(D)** Module-trait associations were evaluated by correlations between module eigengenes and sample traits. **(E)** Scatterplot of Gene Significance for RIF vs. Module Membership in five modules.

Co-expression network was constructed by WGCNA. When soft-threshold power was 5 (scale free R2 > 0.85) and the height was 0.25, 15 modules were obtained (Figures 6A–C). Figure 6D revealed relationships between modules and traits. Combined with scatter plots, we identified 5 modules associated with clinical traits: brown module (cor = 0.41, *p* = 3.2e-19), black module (cor = 0.49, *p* = 1.7e-07), pink module (cor = 0.51, *p* = 6.2e-07), turquoise module (cor = 0.44, *p* = 2.8e-112), and red module (cor = 0.8, *p* = 2e-34) (Figure 6E).

### Selection of candidate genes

**FIGURE 7:**
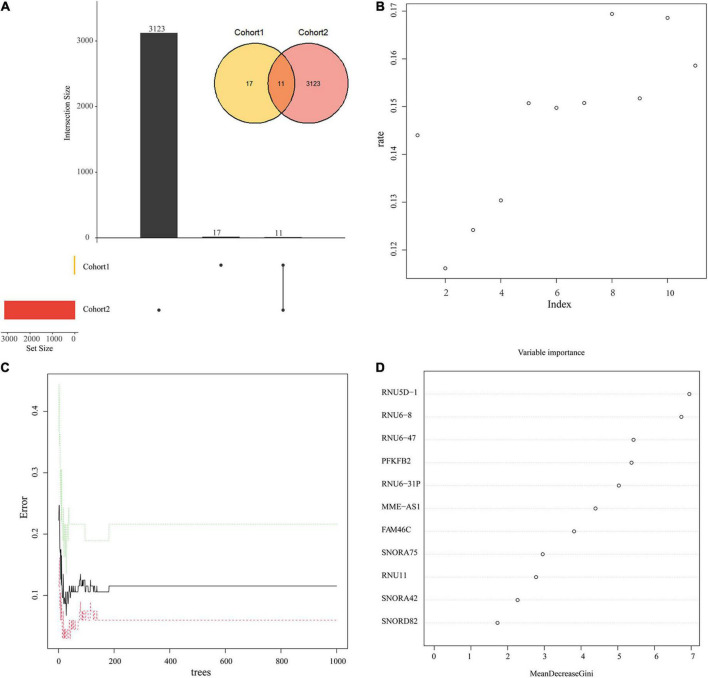
Random Forest model to screen candidate genes. **(A)** The intersection of Cohort 1 and Cohort 2. Cohort 1 (yellow) represents the total number of DEGs, Cohort 2 (red) represents the total number of the above 5 key module genes, and the overlapping part is used for further analysis. **(B)** Scatter plot of the effect of variable number selection on the average error rate. The x-axis represents the number of variables, and the y-axis indicates the out-of-band error rate. **(C)** The influence of the number of decision trees on the error rate. The x-axis represents the number of decision trees, and the y-axis indicates the error rate. When the number of decision trees is approximately 200, the error rate is relatively stable. **(D)** Results of the Gini coefficient method in random forest classifier. The x-axis represents the importance index, and the y-axis indicates the genetic variable.

11 candidate genes were derived from the intersection of DEGs and genes within the five modules (Figure 7A). Next, we input the 11 DEGs into the random forest classifier. The relationship between the model error and the number of decision trees was shown in Figure 7B. It showed it was an optimal parameter when we chose 2 as the parameter of variable number. We chose 200 trees as the parameter of the final model (Figure 7C). After the random forest model was constructed, we selected 10 hub genes whose output variable importance (Gini coefficient) was greater than 2, including *RNU5D-1, RNU6-8, RNU6-47, PFKFB2, RNU6-31P, MME-AS1, FAM46C, SNORA75, RNU11, SNORA42* (Figure 7D). Among these candidate genes, we found that *PFKFB2* is a glycolysis related gene, which is consistent with the energy metabolism altered in the above results.

### Immune landscape associated with the characteristics of old myocardial infarction

**FIGURE 8:**
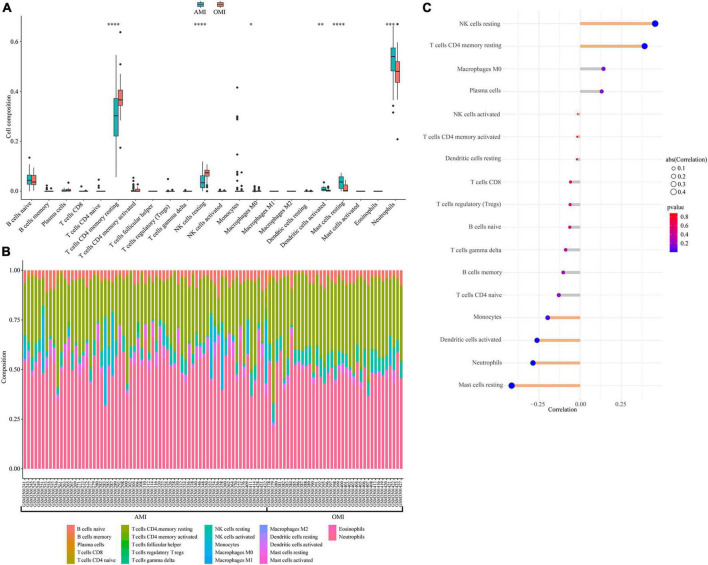
Immune landscape associated with the characteristics of OMI patients. **(A)** Violin plot showing the distribution of 22 immune cells in AMI and OMI patients in GSE123342. **(B)** The relative fraction of 22 types of immunocyte assessed by CIBERSORT. **(C)** The relationship between OMI and distribution of immune cell; yellow: Statistically significant (*P* < 0.05). *P < 0.05, ^**^P < 0.01, ^***^P < 0.005, ^****^P < 0.0001.

Immune-related pathways were obtained in functional enrichment. We further estimated the proportion of 22 immune cell subtypes in peripheral blood using CIBERSORT. The distribution of immune cells was shown in Figure 8A, and T cell CD4 memory resting, NK cells resting, Macrophages M0, Dendritic cells activated, Mast cells resting, and Neutrophils were differentially distributed. Figure 8B showed the proportion of immune cells in each sample. We also analyzed the relationship between immune cell distribution and clinical traits. Among them, NK cells resting (cor = 0.46, *p* = 1.2e-06) and T cell CD4 memory resting (cor = 0.39, *p* = 4.1e-05) were positively correlated with OMI; Mast cells resting (cor =−0.42, *p* = 1.1e-05), Neutrophils (cor =−0.29, *p* = 3.2e-03), Dendritic cells activated (cor =−0.26, *p* = 7.2e-03) and Monocytes (cor =−0.20, p = 4.5e-02) were negatively correlated with OMI (Figure 8C).

### Identification of immune-related gene

We performed Spearman correlation analysis to identify the relationship between 10 hub genes and 6 types of immune cells (Figure 2A). We found that *PFKFB2* was associated with all six types of immune cells. The expression levels of *PFKFB2* in the samples were shown in Figure 2B. *PFKFB2* was significantly downregulated in OMI samples. PFKFB2 is known to be a key regulator of glycolysis. We therefore performed GSEA on two groups of samples (AMI and OMI). The results of GSEA enriched the three energy metabolism pathways of “Galactose metabolism,” “Metabolic pathways,” and “Starch and sucrose metabolism,” all of which were down-regulated (Supplementary Figure 1 and Supplementary Table 2).

### Validation of *PFKFB2* and immune cells in clinical samples

To further verify our analytical results, we detected the expression levels of *PFKFB2* in clinical samples (Supplementary Tables 3, 4). Consistently, *PFKFB2* was significantly downregulated in OMI (Figure 3A). We also collected blood counts from these patients, including neutrophils, basophils, monocytes, lymphocytes, and eosinophils (Figures 3B,C and Supplementary Figure 2 and Supplementary Tables 3, 4). In the available samples, we only detected differential distribution of neutrophils and monocytes between the two types of samples, but no significant differences in lymphocytes. Here, lymphocytes were not divided into various subpopulations. Spearman’s correlation matrix was plotted against data from clinical samples (Figure 3D). *PFKFB2* was shown to be associated with monocytes.

## Discussion

In this study, we combined methods such as Cibersort, WGCNA, and Random Forest Model to identify *PFKFB2* related to glycolysis as a key gene, and measured the differential distribution of immune cells in peripheral blood samples of patients with AMI and OMI. It was found that *PFKFB2* was related to the differential distribution of immune cells. Further, we confirmed the low expression of *PFKFB2* in clinical samples of OMI and the correlation between *PFKFB2* and immune cell counts in clinical samples. This study investigates the characteristic genes from AMI to OMI at the level of peripheral blood transcriptome and found that *PFKFB2* is the key gene in this process, which can reflect the transition from AMI and predict the distribution of immune cells. These findings indicate that *PFKFB2* may be a potential target for early clinical intervention of AMI.

The immune response plays an important role after MI (13). Our study revealed that Dendritic cells activated, Monocytes, Mast cells resting and Neutrophils were inversely associated with OMI, while T cells CD4 memory resting and NK cells resting were positively associated. Indeed, inflammatory responses and immune circulating cells, such as neutrophils and lymphocytes, have been shown to serve as critical mediators involved in the development of coronary heart disease (14). Studies have shown that after AMI, extramedullary monocytes/macrophages are rapidly activated by sympathetic nerve and IL1 signaling, and then recruit neutrophils to the infarct site (15). Neutrophils mediate local inflammation and tissue damage at the infarct site through phagocytosis, release of reactive oxygen species, and degranulation (16). At OMI stage, the inflammatory response to the infarct subsidies (17). This is consistent with the results of our study. We found that in the peripheral blood of OMI, neutrophils decreased. Moreover, monocytes/macrophages can promote angiogenesis and collagen matrix formation, and limit adverse remodeling during infarct healing (18). Räber et al. found that patients who suffered from AMI had fewer macrophages in coronary lesions after receiving long-term standardized treatment (19). In our study, monocytes/macrophages showed a decreasing trend and were associated with clinical features, but there were no significant differences. This may be due to the large heterogeneity between samples and in our subsequent clinical sample validation, we found differences in monocytes. In addition, the depletion of dendritic cells can reduce the number of neutrophils and T cells after MI, thereby reducing the inflammatory response, reducing the infarct area, and ultimately improving cardiac function (20). Taken together, our results suggested that immune cells played an important role in the transition from AMI to OMI.

Through WGCNA and Random Forest Model analysis, we identified 10 key genes, including *RUN5D-1, RUN6-8, RUN6-47, PFKFB2, RUN6-31P, MME-AS1, FAM46C, SNORA75, RUN11, SNORA42*. Among them, *PFKFB2* is a key regulator of glycolysis, showing an important role of metabolism. Spearman correlation analysis was performed between the above 10 genes and 6 types of immune cells, and we found that *PFKFB2* was correlated with the 6 types of immune cells. The main function of *PFKFB2* is to phosphorylate fructose 6-phosphate to fructose 2,6-bisphosphate, which is an allosteric activator of *PFK1*, a key rate-limiting enzyme in sugar degradation (21). There is no doubt that *PFKFB2* affects the activity of the glycolytic process. Myocardial hypoxia can activate the *HIF/AKT* signaling pathway, regulate the increased expression of *PFKFB2*, and then improve cardiac function after MI and reduce cardiomyocyte apoptosis by reprogramming cellular glycolysis (22). At the same time, some researchers found that activated neutrophils and M1 macrophages in myocardial tissue during AMI showed the characteristics of enhanced glycolysis (23, 24). However, the role of *PFKFB2* in peripheral blood has not been reported so far. Before this study, we still did not know the expression characteristics and role of *PFKFB2* in the peripheral blood between AMI and OMI. Our results showed that *PFKFB2* was lowly expressed in the peripheral blood of OMI, and the peripheral blood metabolic level was down-regulated.

Besides, some researchers had found that *PFKFB2* and its isozyme could affect the immune cell chemotaxis and distribution by affecting glycolysis. Poels et al. suggested that inhibition of the glycolytic process could reduce inflammation (25). Zhang et al. also applied *PFKFB2* to predict the level of immune cell (26). All of these suggested an association between *PFKFB2* and immune cells.

Further, we additionally collected clinical samples to validate our results. We obtained independent clinical cohort of the blood routine results of AMI (*n* = 81) and OMI (*n* = 101) patients treated in our hospital in the past 6 months (Supplementary Table 5), and the down-regulation of neutrophils and monocytes in OMI was verified (Figure 4). Lymphocytes were not subtyped and no meaningful results were found. To further explore the expression characteristics of *PFKFB2*, we also studied on its expression level in patients with UA and no previous history of MI, we found that the expression of *PFKFB2* in UA was similar to that in AMI, with no significant difference, but higher than that in OMI (Supplementary Figures 3A,B and Supplementary Tables 3, 4). This reflected *PFKFB2* plays a key role in the pathophysiological development after MI.

Our study also had certain limitations. Firstly, the immune status after MI can be affected by multiple variables such as aging, smoking, personal stress, and genetic background, involving complex mechanisms (27, 28), which requires us to set strict inclusion and exclusion criteria, expand the sample size, and reduce data bias and error. Second, we were unable to obtain the expression of *PFKFB2* for each immune cell subtype. This needs to be further explored by single-cell sequencing.

## Conclusion

In conclusion, our findings suggested that *PFKFB2* is a key gene that reflects the transition from AMI to OMI, and predicts the level of immune cells, which may be a potential therapeutic target for early clinical intervention of AMI, providing a new perspective for improving myocardial fibrosis and adverse remodeling.

## Data availability statement

The datasets presented in this study can be found in online repositories. The names of the repository/repositories and accession number(s) can be found in the article/Supplementary material.

## Ethics statement

The studies involving human participants were reviewed and approved by the Ethics Committee of Xiangya Hospital, Central South University. The patients/participants provided their written informed consent to participate in this study.

## Author contributions

JL: conceptualization, methodology, data curation, formal analysis, and writing—original draft. XY: investigation, validation, data curation, formal analysis, and writing—original draft. QM: project administration and writing—review and editing. XH, YZ, YK, YL, CL, HG, LM, and JT reviewed the manuscript. All authors contributed to the article and approved the submitted version.
