# Supplementary material for: Identification of PFKFB2 as a key gene for the transition from acute to old myocardial infarction in peripheral blood
Source: Front Cardiovasc Med. 2022 Dec 6;9:993579. doi: 10.3389/fcvm.2022.993579 (PMC9763698; doi:10.3389/fcvm.2022.993579)
Supplement: Supplementary file 1 [file Data_Sheet_1.docx]

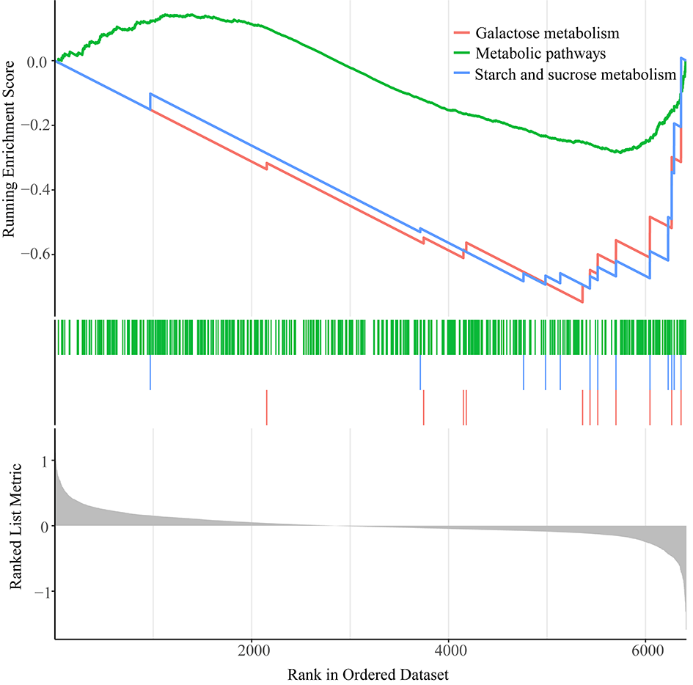
FIGURE S1|GSEA on two groups of samples (AMI and OMI).

FIGURE S2| The distribution of basophil counts (A), eosinophil counts (B), and lymphocyte counts (C) in AMI and OMI is shown, respectively (1*10^9).


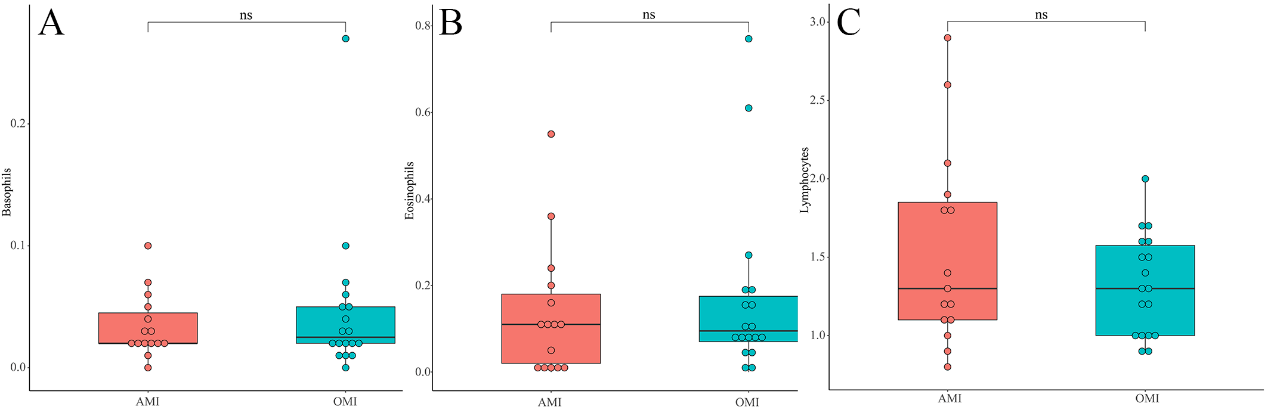


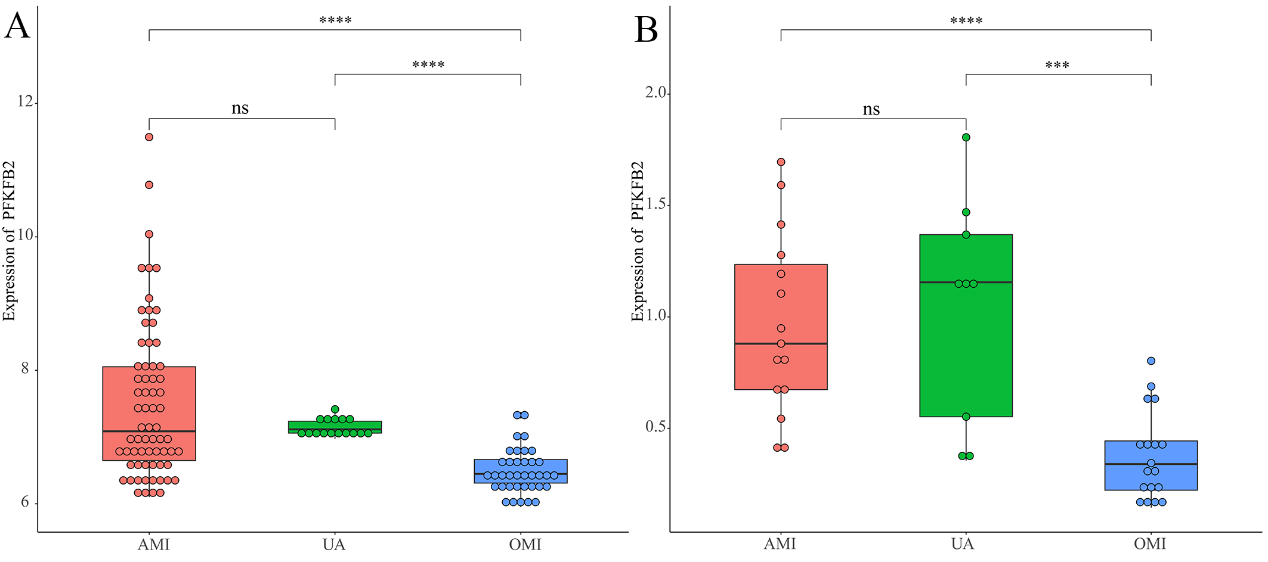
FIGURE S3| Expression of PFKFB2 in AMI, UA and OMI. (A) Comparison of three types (AMI, UA, and OMI) of samples in GSE123342. (B) Comparison of three types (AMI, UA, and OMI) of clinical samples.

The sequencing data of unstable angina (UA, n=16; clinical symptoms of cardiac ischemia without myocardial necrosis) is from GSE29111. The analysis method is consistent with the description in the manuscript.
